# Repurposing FDA-approved drugs as inhibitors of therapy-induced invadopodia activity in glioblastoma cells

**DOI:** 10.1007/s11010-022-04584-0

**Published:** 2022-10-27

**Authors:** Dylan Jones, Clarissa A. Whitehead, Marija Dinevska, Samuel S. Widodo, Liam M. Furst, Andrew P. Morokoff, Andrew H. Kaye, Katharine J. Drummond, Theo Mantamadiotis, Stanley S. Stylli

**Affiliations:** 1 Level 5, Clinical Sciences Building, Department of Surgery, The University of Melbourne, The Royal Melbourne Hospital, Parkville, VIC 3050 Australia; 2 Department of Neurosurgery, The Royal Melbourne Hospital, Parkville, VIC 3050 Australia; 3 Department of Microbiology and Immunology, School of Biomedical Sciences, The University of Melbourne, Parkville, VIC 3010 Australia; 4 Hadassah University Medical Centre, 91120 Jerusalem, Israel

**Keywords:** Glioblastoma, Invadopodia, Invasion, Temozolomide, Radiotherapy, Drug repurposing

## Abstract

Glioblastoma (GBM) is the most prevalent primary central nervous system tumour in adults. The lethality of GBM lies in its highly invasive, infiltrative, and neurologically destructive nature resulting in treatment failure, tumour recurrence and death. Even with current standard of care treatment with surgery, radiotherapy and chemotherapy, surviving tumour cells invade throughout the brain. We have previously shown that this invasive phenotype is facilitated by actin-rich, membrane-based structures known as invadopodia. The formation and matrix degrading activity of invadopodia is enhanced in GBM cells that survive treatment. Drug repurposing provides a means of identifying new therapeutic applications for existing drugs without the need for discovery or development and the associated time for clinical implementation. We investigate several FDA-approved agents for their ability to act as both cytotoxic agents in reducing cell viability and as ‘anti-invadopodia’ agents in GBM cell lines. Based on their cytotoxicity profile, three agents were selected, bortezomib, everolimus and fludarabine, to test their effect on GBM cell invasion. All three drugs reduced radiation/temozolomide-induced invadopodia activity, in addition to reducing GBM cell viability. These drugs demonstrate efficacious properties warranting further investigation with the potential to be implemented as part of the treatment regime for GBM.

**Supplementary Information:**

The online version contains supplementary material available at 10.1007/s11010-022-04584-0.

## Introduction

Gliomas are the most common primary central nervous system (CNS) malignancy in adults, accounting for approximately 80% of all CNS malignancies [1], with an average annual age-adjusted incidence of 6.0 per 100,000 population and a 5 year survival rate of only 5% in the United States between 2010 to 2014 [2]. The most common and aggressive form of glioma, glioblastoma (GBM), accounts for 61% of all gliomas [2]. Despite multimodal treatment with surgery, fractionated radiotherapy (RT) and temozolomide (TMZ) chemotherapy, the median survival for GBM patients is only 6–15 months [3, 4].

Gliomas exhibit several classic tumour cell characteristics, including genomic instability, resistance to apoptosis, proliferation, and invasion [5]. GBM is highly invasive, with widespread infiltration of tumour cells into surrounding normal brain parenchyma. This inevitable spread prevents complete surgical tumour resection, with residual post-operative tumour cells contributing to recurrence. In addition, the development of resistance to TMZ and RT in residual cells contributes to tumour recurrence and patient death.

It has been previously shown that invadopodia, dynamic, actin-rich membrane-localized structures, facilitate cancer cell invasion, including in GBM [6, 7]. These specialized structures extend into and adhere to the surrounding extracellular matrix (ECM) and proteolytically degrade ECM substrates using various transmembrane proteases, including membrane type 1-matrix metalloproteinase (MT1-MMP), and secreted proteases such as matrix-metalloproteinase (MMP)-2 and MMP-9 [8–10]. Importantly, the expression of MMP-2 and MMP-9 is upregulated in GBM [11, 12]. Invadopodia are found in glioma cell lines and tumour cells isolated from human GBM tissue, suggesting a role in glioma invasion [7, 13]. We have also previously shown that the invadopodia regulator, Tks5, correlates with glioma patient survival [14], further supporting the role of invadopodia in glioma.

Ionizing radiation is an important modality in cancer treatment, with a survival benefit for many cancers [15], including glioma [16]. However, it has been proposed that RT may exacerbate the invasive and migratory behaviour of cancer cells [17, 18]. Studies have demonstrated that RT may facilitate tumour invasion through treatment-induced secretion of pro-invasive factors such as the MMP family [19–23]. As well as promoting invasion, an increase in MMP-2 secretion may also assist tumour survival by reducing apoptosis, proliferation and angiogenesis [24]. This is an important consideration, as the majority of GBM inevitably recur in the vicinity of the target volume of RT around the surgical resection cavity [25]. This is supported by several studies which report an increase in the migratory and invasive potential of GBM cells that survive RT and TMZ treatment [26–29].

The most recent advance in GBM treatment, in 2005, was the discovery that complementing surgical resection and RT with TMZ treatment marginally increased survival rates [30]. With no major advances since this discovery, GBM patients continue to face a poor prognosis, highlighting the need for innovative and effective therapeutic strategies. Drug repurposing, finding new uses, for existing drugs approved for other indications, is gaining momentum, particularly as the pipeline for drug discovery involves significant financial investment, and a timeframe of 10 to 15 years [31]. Drug repurposing benefits from the existing knowledge on dosage, safety, and side effects, negating the need for phase I and II trials and ultimately reducing both lead time and cost. Therefore, we examined several FDA-approved drugs for their potential to impair invadopodia activity in GBM cells.

## Materials and methods

### FDA-approved drugs

The 20 FDA-approved drugs used in this study (Supp. Table 1) were supplied by Selleckchem (Selleckchem, Houston, TX, USA) at a concentration of 10 mM in DMSO and were stored at − 80 °C. They were derived from a larger commercial library (Catalogue Number: L1300) and were chosen for potential impact on invadopodia activity, based on involvement of drug gene targets in cancer cell invasion and expression in GBM. The ideal drug would have a dual effect in reducing both cell viability and invadopodia activity in GBM cells surviving RT and TMZ treatment.

### Cell lines and culture

U87MG and LN229 cell lines were purchased from the ATCC (American Type Culture Collection). MU41 is a patient-derived cell line obtained from an explant human GBM biopsy at The Royal Melbourne Hospital (Melbourne Health Research Ethics Approval Number 2009.116). The cells were cultured in DMEM (Life Technologies) supplemented with 10% (vol/vol) heat-inactivated fetal bovine serum (HyClone), penicillin (100 U/ml), and streptomycin (10 μg/ml). All cells were maintained in a humidified atmosphere of 10% CO_2_ at 37 °C and used within the first 20 passages.

### Western blot analysis

Western blot analysis of GBM cell protein lysates (20 μg) was performed using NuPage 4%-12% bis–tris precast gels (Invitrogen) and transferred onto nitrocellulose blotting membrane (GE Healthsciences). The membranes were blocked with 3% bovine serum albumin in 1% TBST for 1 h prior to overnight incubation with primary antibodies at 4 °C. The following antibodies were utilized for this study: GAPDH (diluted 1:1000, Cat No. 14C10, Cell Signalling Technologies), N-WASP (diluted 1:1000, Cat. No. SC271484, Santa Cruz Biotechnology); Nck1 (diluted 1:1000, Cat No. 15B9, Cell Signaling Technologies), Cortactin (diluted 1:1000, Cat No. SC555888, Santa Cruz Biotechnology) and Tks5 (diluted 1:1000, Cat No. SC30122, Santa Cruz Biotechnology). Subsequently, the membranes underwent three 5 min washes in 1 × TBST and then incubated with the secondary antibody (1:10,000; Cat. No. 1706515, anti-rabbit; Cat. No. 1706516, anti-mouse, BioRad) and developed using an enhanced chemiluminescence reagent (GE Healthcare) and exposure onto SuperRX x-ray film (Fujifilm).

### Gelatin zymography

For zymographic analysis of cell culture medium, 1 × 10^6^ cells were seeded per well in six well plates (Corning) and were allowed to adhere overnight before washing with sterile phosphate buffered saline (PBS) and further incubation in 2 ml of serum-free OptiMem® (Thermofisher Scientific) for 24 h. 200 μl aliquots of the conditioned OptiMem® medium was then sampled and centrifuged at 1000×*g* (4 °C) for 10 min before storage at − 80 °C. NuPAGE precast gels (Invitrogen, Australia) were used for the gelatin based zymography and the conditioned OptiMem® media samples were normalized against the corresponding cell protein lysate concentration, as determined using the Bicinchonic acid (BCA) protein assay (Pierce, Thermofisher Scientific). Separation of the media samples was performed by electrophoresis at 125 V for 1.5 h in 1 × Novex Tris–Glycine SDS Running Buffer. Subsequently, the gels were then removed and incubated for 30 min at room temperature in 1 × Novex zymogram renaturing buffer (Thermofisher Scientific), followed by a 30 min incubation at room temperature in 1 × Novex zymogram developing buffer. This was then replaced with new developing buffer for an overnight incubation at 37 °C. The gels were then washed in distilled water and stained for 1 h in Simply Blue Stain (Life Technologies) followed by additional washing in distilled water until clear gelatinolytic bands were visible. Identification of the band molecular weight was determined using loaded Precision Blue protein markers (Bio-Rad) and the gels were scanned using a flatbed scanner.

### Cell viability assays

Cells were seeded (1 × 10^4^ cells/well) into 96 well plates and were allowed to adhere overnight. The cells were then treated with the FDA-approved drugs listed in Supplementary Table 1 at increasing concentrations (0, 0.01, 0.1, 1 and 10 μM) in triplicate for 72 h in a humidified incubator (10% CO_2_ at 37 ℃). Following the incubation, cell viability was assessed with a plate reader (absorbance—570 nm wavelength) using a CellTiter 96 Non-radioactive Cell Proliferation Assay (MTT) (Promega), as per the manufacturer’s instructions.

### Invadopodia matrix degradation assay

FITC-gelatin invadopodia matrix assays were performed as previously described [32]. The basal invadopodia activity of the cell lines was determined by seeding 1 × 10^5^ cells per FITC-gelatin coated coverslip for 24 h in a humidified incubator (10% CO_2_ at 37 °C). The cells were then then washed with 1 × PBS and fixed in 4% paraformaldehyde for 15 min. The cells were permeabilized with 0.2% Triton-X-100 and then stained with rhodamine phalloidin (invadopodia actin puncta) and DAPI (nucleus), and the coverslips were mounted with Vectashield (Vector Laboratories). Images were then acquired using a Nikon A1 + confocal microscope system utilizing a Plan Apo VC 60 × Oil DIC N2 immersion objective. Degraded gelatin was defined as black areas depleted of fluorescent gelatin within each image. A total of 10 random image fields were acquired for each sample. Images were subsequently analyzed using ImageJ (version 1.51n), and threshold and region tools were utilized to define the total region of degradation present within each acquired image field. A particle counter macro was then employed to calculate the total area of FITC-conjugated gelatin degradation, and this was then standardized relative to the number of DAPI-positive cells that were present within the image field. The LN229 cells were irradiated at 2 Gy and after 24 h (10% CO_2_ at 37 °C), the cells were treated with 50 μM of TMZ for a further 24 h (10% CO_2_ at 37 °C). The cells were then incubated with 0.01 μM of the FDA-approved drugs for an additional 72 h (10% CO_2_ at 37 °C). The cells were then trypsinized and seeded at a density of 1 × 10^5^ cells per FITC-gelatin coated coverslip for 24 h in the absence of drug (10% CO_2_ at 37 °C) prior to fixing and staining as previously outlined.

### In vitro scratch wound assay

LN229 cells were seeded into 6-well plates (3 × 10^5^ cells/well) and allowed to adhere overnight in a humidified environment (10% CO_2_ at 37 °C). The cells were then incubated in the presence of drug (0.01 μM) for 72 h, after which 5 μg/ml of Mitomycin C (2 h, 10% CO_2_ at 37 °C) was then added to the cells to arrest proliferation, followed by the introduction of a scratch 2 h later. The cells were then washed twice in PBS before fresh medium was added. Images were acquired at 0, 6 and 24 h using a 4 × objective. Images were analysed using Image J (Version 1.51n) to define the area of the wound.

### Cultrex BME invasion assay

Cells were seeded (2.5 × 10^4^ cells/well) in 24 well plates, allowed to adhere overnight before the addition of serum-free Optimem (24 h). Membrane inserts were then coated with a 100 μl volume of 0.5× BME as per the manufacturer’s protocol. GBM cells were treated with RT/TMZ and the candidate drugs as outlined in the invadopodia assay, prior to seeding in the top chamber of the BME coated membranes.

### Datamining

Differential mRNA expression levels of invadopodia regulators in glioma tissue were retrieved from the Oncomine™ v4.5 database (www.oncomine.org; Compendia Bioscience™, Ann Arbor, MI, USA, part of Life Technologies). Oncomine™ is an online cancer microarray database containing 715 datasets (86,733 samples) compiled from various cancer studies. The threshold for the inclusion of data analysis was set to *p* < 0.05 for significance and an mRNA expression fold difference of > 2. All data are log transformed, and the standard deviation is normalized to one per array studied.

The mRNA expression levels of the FDA-approved candidate drugs target genes (bortezomib, fludarabine and everolimus) in glioma samples of different grades from The Cancer Genome Atlas (TCGA) and Chinese Glioma Genome Atlas (CGGA) cohorts were analysed using the Gliovis glioma data portal (http://gliovis.bioinfo.cnio.es/). A GBM patient survival analysis was also conducted using the Glioblastoma Bio Discovery Portal (GBM-BioDP; https://gbm-biodp.nci.nih.gov/), which is an online resource (https://gbm-biodp.nci.nih.gov) for examining The Cancer Genome Atlas (TCGA) data associated with GBM [33]. GBM-BioDP enables the probing of gene expression profiles based on known molecular subtypes and association with clinical outcomes [34]. Data sourced from GBM-BioDP was used to demonstrate that co-expression of invadopodia genes with the gene targets of the candidate FDA-approved drugs portended a poorer prognosis compared to the invadopodia genes or drug target genes alone.

### STRING database: protein–protein interaction analysis

The Search Tool for the Retrieval of Interacting Genes/proteins (STRING V11.5; www.string-db.org) [32], is a database that searches for known protein interactions online. The minimum required interaction score was set with a medium confidence of (0.400) and a network interaction map of the drug target genes and invadopodia genes was constructed (Supp. Figure 2).

### Statistical analysis

Statistical significance was determined using an unpaired, unequal variance, two-tailed t-test with the use of GraphPad Prism 7 (Prism 7.00 for Windows, GraphPad Software, La Jolla, CA, USA). Values were considered statistically significant if *p* < 0.05.

## Results

### Functional matrix degrading invadopodia are present in GBM cell lines

**Fig. 1:**
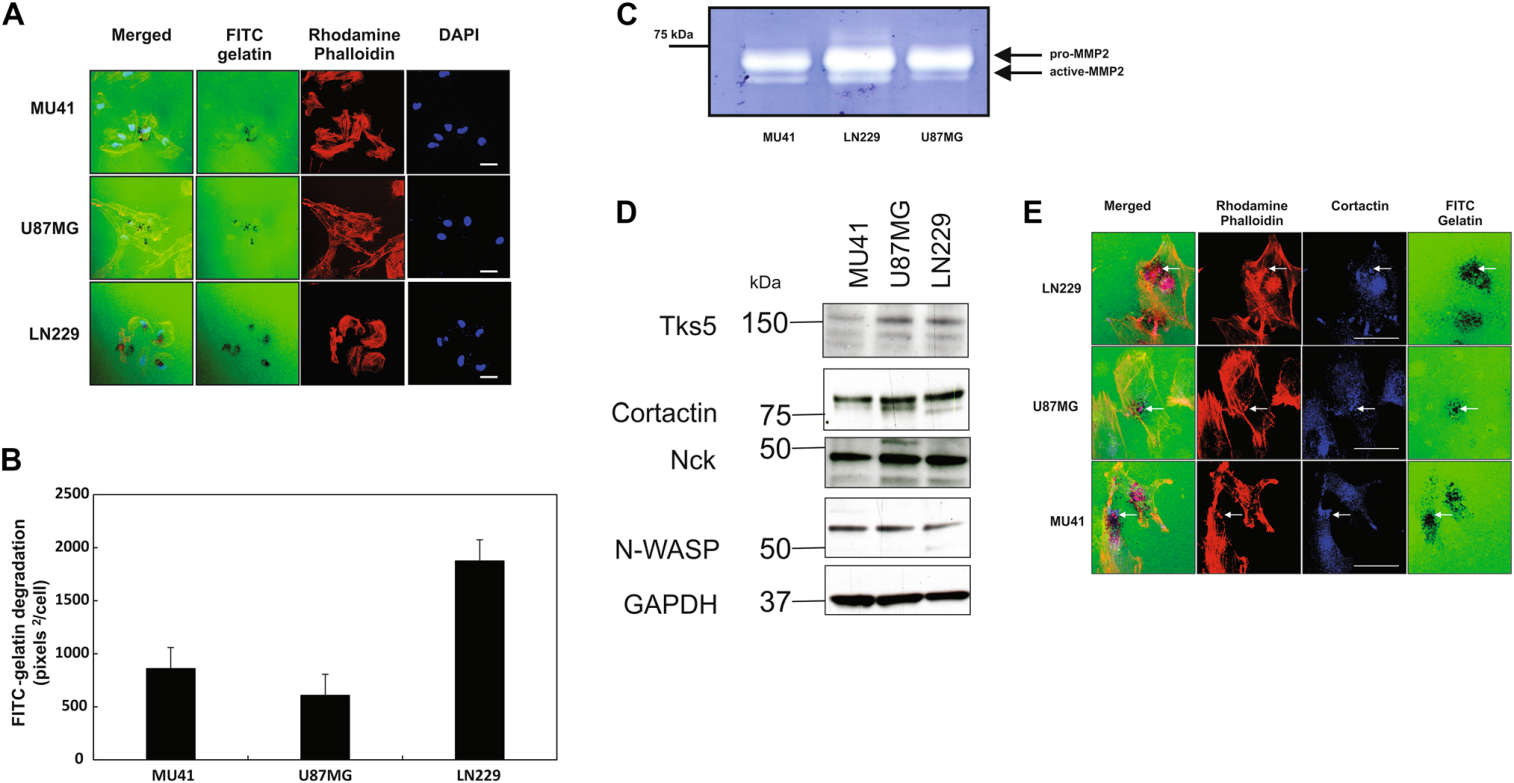
GBM cell lines form functional invadopodia and secrete MMP-2. **a** MU41, U87MG and LN229 GBM cells were seeded on cross-linked FITC-gelatin (24 h) to detect the presence of FITC-gelatin degrading invadopodia. White scale bars represent 20 μM. Degraded areas of FITC-labelled gelatin are evident as black areas devoid of FITC-labelled gelatin (*green*). DAPI staining of the nucleus is shown in blue, and rhodamine-conjugated phalloidin was used to stain for actin filaments and actin puncta (invadopodia). **b** Graph depicting the basal invadopodia-mediated FITC-gelatin degradation activity of the GBM cell lines in (**a**). Experiment was repeated three times. **c** Gelatin zymography analysis showing MMP-2 activity at 24 h after incubation of GBM cells in serum-free Optimem®. The experiment was repeated three times and a representative image is shown. **d** Western immunoblot analysis of various regulators of invadopodia formation/activity in the listed GBM cell lines. **e **Endogenous cortactin colocalizes with invadopodia actin puncta. GBM cells were seeded on cross-linked FITC-labelled gelatin. After 24 h, the cells were fixed and stained for actin filaments with rhodamine phalloidin (*red*), cortactin primary antibody and an Alexa 405 secondary antibody (*blue*). The white arrows indicate co-localization of rhodamine phalloidin-stained actin puncta with cortactin within invadopodia. The experiment was repeated twice and representative images are shown. Scale bar = 20 μM

The three cell lines used in this study (MU41, U87MG and LN229) form functional invadopodia that degrade the FITC-labelled gelatin which can be seen to co-localize with rhodamine phalloidin-stained actin puncta (Fig. 1a). The presence of functional invadopodia is further supported with the observed co-localization of cortactin with actin puncta (Fig. 1e). Quantification revealed that LN229 cells exhibited the highest level of invadopodia-mediated FITC-gelatin degrading activity (Fig. 1b). Importantly, zymographic analysis of serum-free conditioned medium isolated from the cell lines revealed that the prominent MMP forms detected were pro-MMP-2 (72 kDa) and active MMP-2 (65 kDa) (Fig. 1c).

To support the functional results indicating the presence of invadopodia in our GBM cell lines, we determined the expression levels of several known regulatory invadopodia-related proteins including Tks5, cortactin, Nck, MMP-2 and N-WASP using western blotting (Fig. 1d). All three cell lines showed expression of these invadopodia-related proteins, although levels varied. LN229 cells displayed both the highest invadopodia-mediated FITC-gelatin degradation and highest expression of invadopodia-associated proteins.

### Repurposed FDA-approved drugs can reduce GBM cell line viability

**Fig. 2:**
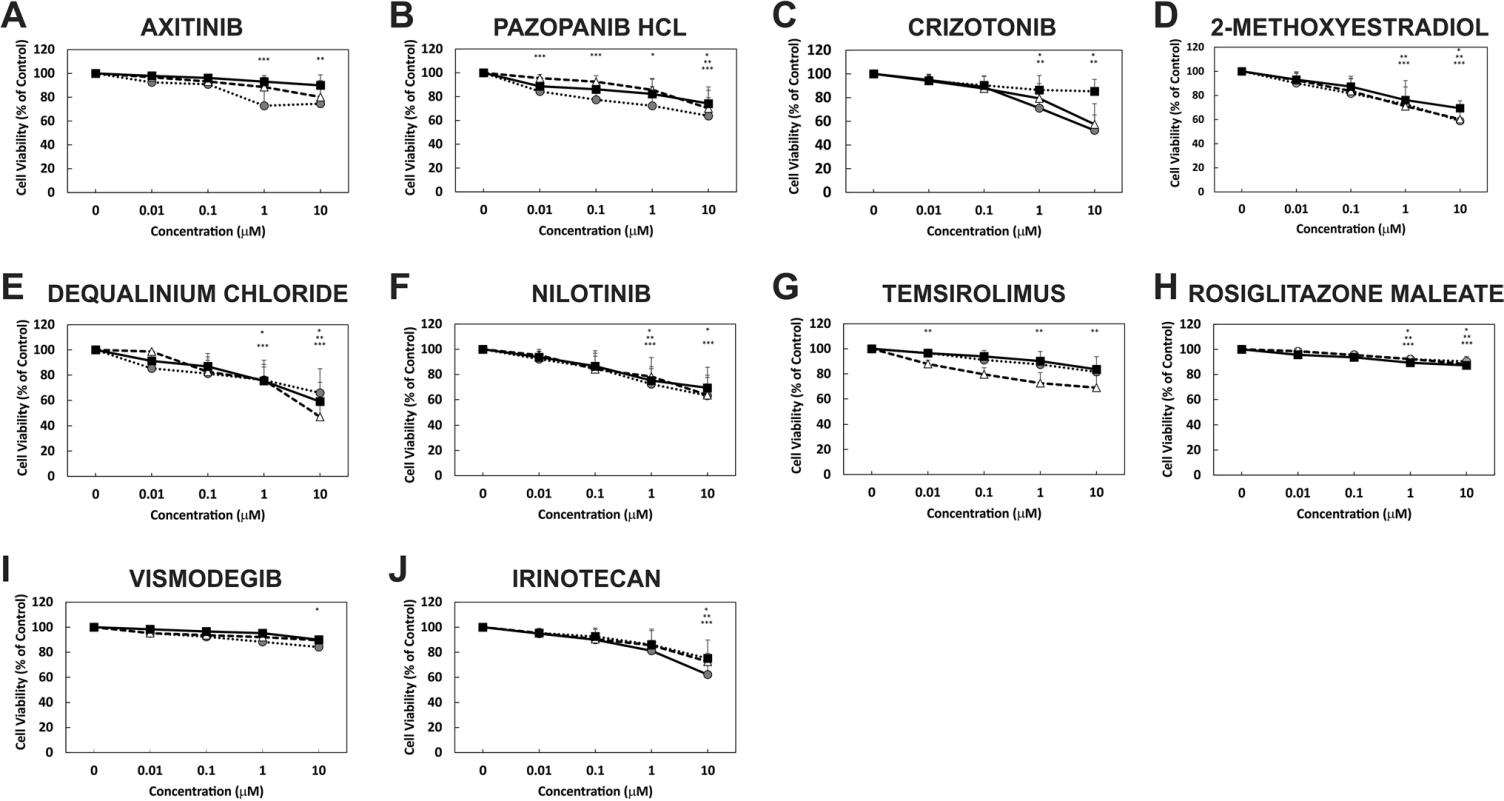
FDA-approved drugs reduce GBM cell line viability. GBM cell lines MU41 (*Black*), LN229 (*Grey*) and U87MG (*white*) were incubated at several concentrations (0, 0.01, 0.1, 1, and 10 μM) of the FDA-approved drugs for 72 h before cell viability was determined using an MTT assay. Cell viability is represented as percentage survival relative to untreated control cells (*n* = 6 experiments; mean + / − standard deviation. *(MU41), **(LN229), ***(U87MG) *P* < 0.05 when compared to the respective control). **a** Axitinib, **b** Pazopanib HCl, **c** Crizotinib, **d** 2-methoxyestradiol, **e** Dequalinium Chloride, **f** Nilotinib, **g** Temsirolimus, **h** Rosiglitazone Maleate, **i** Vismodegib, **j** Irinotecan.

**Fig. 3:**
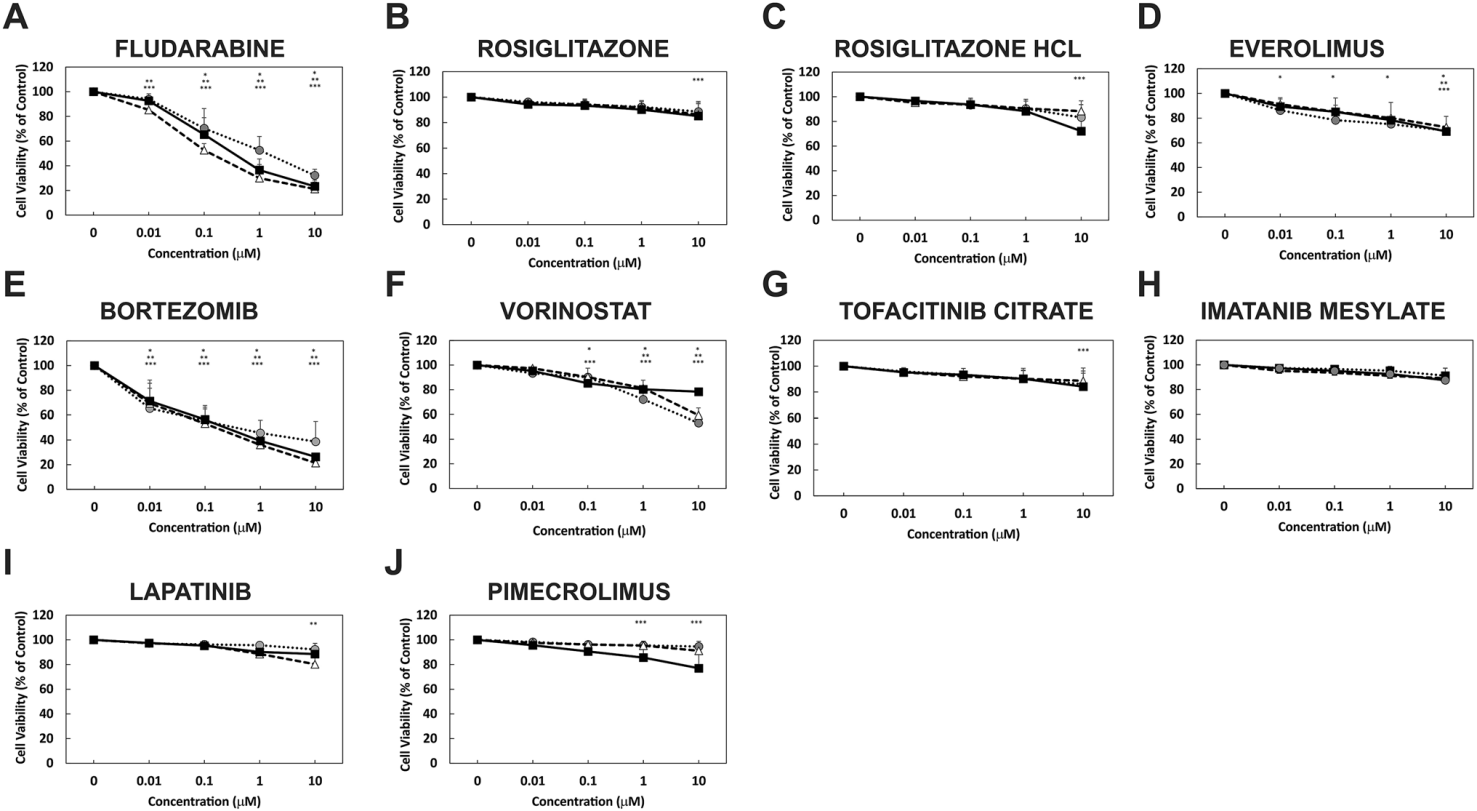
FDA-approved drugs reduce GBM cell line viability. GBM cell lines MU41 (*Black*), LN229 (*Grey*) and U87MG (*white*) were incubated at several concentrations (0,0.01, 0.1, 1, and 10 μM) of the FDA-approved drugs for 72 h before cell viability was determined using an MTT assay. Cell viability is represented as percentage survival relative to untreated control cells (*n* = 6 experiments; mean ± standard deviation. *(MU41), **(LN229), ***(U87MG) *P* < *0.05* when compared to the respective control). **a** Fludarabine, **b** Rosiglitazone, **c** Rosiglitazone HCl, **d** Everolimus, **e** Bortezomib, **f** Vorinostat, **g** Tofacitinib citrate, **h** Imatinib mesylate, **i** Lapatinib, **j** Pimecrolimus.

The twenty FDA-approved drugs (Supp. Table 1) were first examined for their ability to reduce GBM cell viability. A variable response to the drugs over the concentration range examined was observed (Figs. 2, 3). Drugs were then ranked based on efficacy across multiple concentrations and the number of cell lines affected (Supp. Table 2). Three candidate drugs were then selected for further analysis including bortezomib, everolimus, and fludarabine. Bortezomib was the most effective at reducing GBM cell viability across all lines.

### Bortezomib reduces GBM cell migration, whereas bortezomib, everolimus and fludarabine reduce invadopodia-mediated FITC-gelatin degradation in LN229 cells

**Fig. 4:**
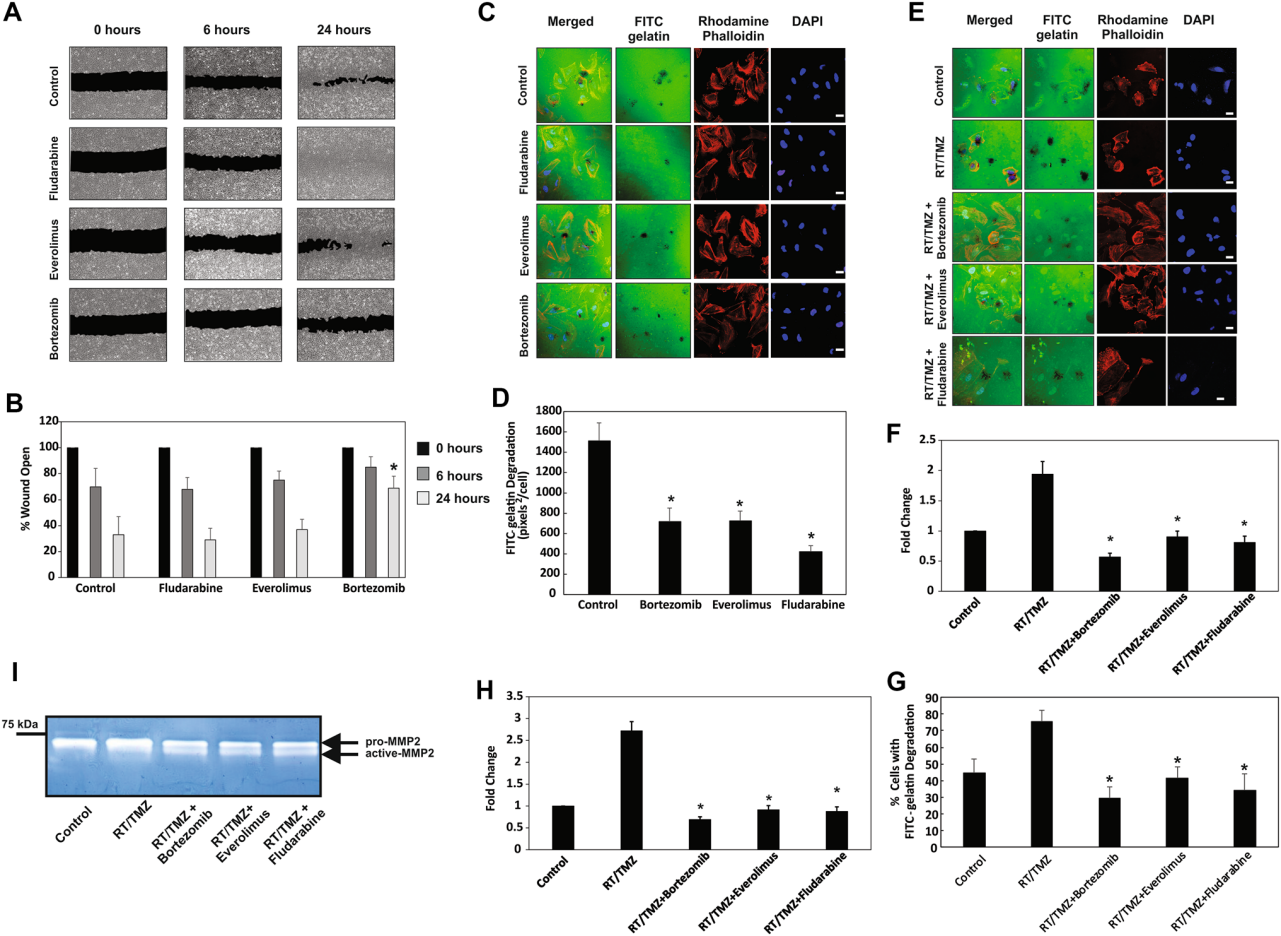
Bortezomib, Everolimus and Fludarabine can reduce invadopodia activity and bortezomib reduces GBM cell migration. **a, b** LN229 GBM cells were treated with 0.01 μM bortezomib, everolimus or fludarabine for 72 h followed by 5 μg/ml mitomycin C for 2 h. A wound was introduced into the confluent monolayer and images were acquired at 0, 6 and 24 h. The wound area for each time point was determined relative to the wound at *t* = 0 h. *P < *0.05* versus control. The experiment was repeated three times and representative images are shown. **c, d** LN229 GBM cells were treated with 0.01 μM bortezomib, everolimus or fludarabine for 72 h and prior to being seeded on cross-linked FITC-gelatin (24 h). The cells were subsequently fixed and stained for actin filaments with rhodamine-conjugated phalloidin (*red*) and DAPI nuclear staining (*blue*). Degraded areas of FITC-labelled gelatin are evident as *black* areas devoid of FITC-labelled gelatin (*green*). The mean FITC-labelled gelatin degrading activity was determined. *P < 0.05 versus control. The experiment was repeated three times and representative images are shown. Scale bar = 20 μm. Bortezomib, Everolimus and Fludarabine can reduce radiotherapy- and temozolomide-induced invadopodia-mediated activity and MMP-2 secretion in LN229 GBM cells. **e–g** LN229 GBM cells were treated with RT/TMZ (2 Gy/50 μM), 24 h prior to the addition of 0.01 μM bortezomib, everolimus or fludarabine for 72 h prior to being seeded on cross-linked FITC-gelatin. After an additional 24 h, the cells were subsequently fixed and stained for actin filaments with rhodamine phalloidin (*red*) and DAPI nuclear staining (*blue*). Degraded areas of FITC-labelled gelatin are evident as *black* areas devoid of FITC-labelled gelatin (*green*). The mean FITC-labelled gelatin degrading activity was determined. *P < 0.05 versus control. The experiment was repeated three times and representative images are shown. Scale bar = 20 μm. **h** Candidate drug treatment reduces LN229 GBM cell invasion through a reconstituted basement membrane. Cells prepared for **e–g** were also used to assess the impact of treatment on invasion through a 3D matrix. The assay provides a fluorescent output proportional to the number of cells that invade through the BME coated membrane. The control group was assigned a relative value of ‘1’ and treatment groups calculated relative to this value. *P < 0.05 versus control. Each group was prepared in triplicate and the experiment repeated three times. **i** Gelatin zymography based analysis shows that candidate drug (bortezomib, everolimus and fludarabine) treatment (0.01 μM) of LN229 GBM cells results in a partial reduction in MMP-2 secretion

As LN229 cells exhibited the highest level of invadopodia activity, we investigated the impact of the three drugs on this activity in LN229 cells. Invadopodia regulators such as cortactin are known to exhibit dynamic interactions with the actin cytoskeleton to regulate cell movement and therefore we also examined the effect of the drugs on cell migration (Fig. 4a, b). Only bortezomib was observed to reduce LN229 cell migration, however, all three drugs significantly reduced the basal levels of invadopodia-mediated FITC-gelatin degradation as seen in the untreated controls (Fig. 4c, d).

### Bortezomib, everolimus and fludarabine can reduce RT and TMZ treatment-induced invadopodia activity in LN229 cells

As we had determined that bortezomib, everolimus and fludarabine could reduce invadopodia-mediated FITC-gelatin degradation in LN229 cells, we also examined whether this ‘anti-invadopodia’ effect could be replicated in GBM cells pre-treated with RT and TMZ. LN229 cells treated with RT/TMZ resulted in approximately a two-fold increase in FITC-gelatin degradation, compared to untreated cells (Fig. 4e, f). However, all three drugs reduced RT/TMZ-induced invadopodia activity and the percentage of cells co-localizing with degraded FITC-gelatin (Fig. 4g). Supporting the reduction in invadopodia activity was an observed functional decrease in the invasive capacity of drug treated LN229 cells through the ECM matrix of an invasion assay (Fig. 4h). Furthermore, zymographic examination of conditioned medium isolated from treated LN229 cells showed that the three drugs also partially reduced MMP-2 secretion (Fig. 4i).

### Invadopodia regulator mRNA expression is increased in glioma

**Fig. 5:**
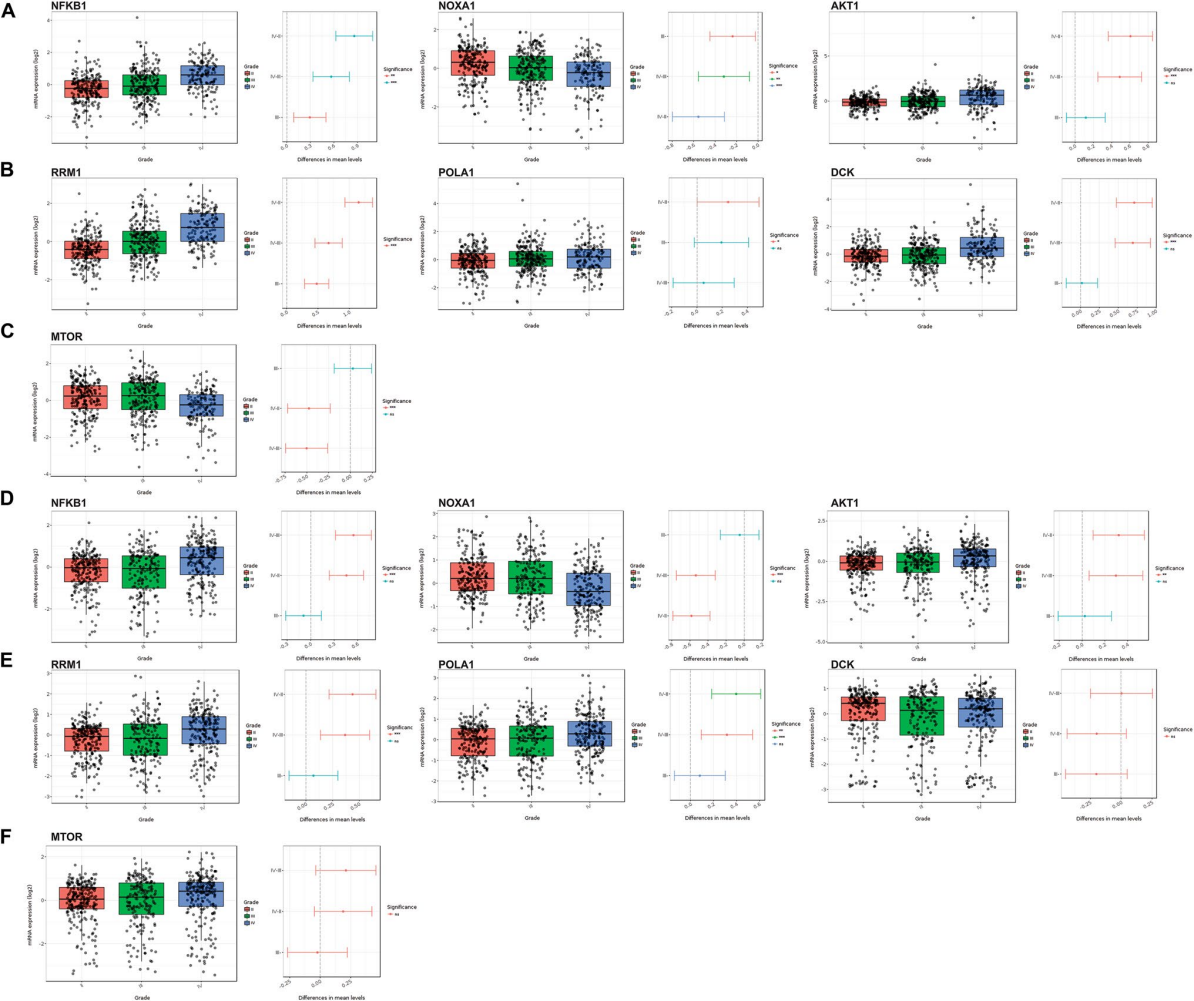
Candidate FDA-approved drug gene targets are generally more highly expressed in GBM compared to lower grade gliomas. Glioma patient samples from The Cancer Genome Atlas (TCGA) (**a**–**c**) and the Chinese Glioma Genome Atlas (CGGA) (**d**–**f**) were analyzed using the GlioVis glioma data portal to examine the gene targets for the candidate FDA-approved drugs (Bortezomib—NFKB1, NOXA1, AKT1; Fludarabine—RRM1, POLA1, DCK; Everolimus—MTOR). Glioma grades are shown on the x-axis (grades II, III, IV). Statistical significance (Tukey’s Honest Significant Difference) is indicated based on differences in mean mRNA expression levels, *p < 0.05, **p < 0.005, ***p < 0.0005, *NS* not significant

Gene expression datasets were examined from seven independent glioma studies showing a significant increase in mRNA levels of invadopodia regulators in GBM tissue compared to normal brain (Table 1), which was also observed for the candidate drug gene targets (Table 2). Analysis of the TCGA and CGGA datasets revealed that gene targets of the three drugs were generally more highly expressed in grade IV glioma (GBM) (Fig. 5). We also utilized GBM-BioDP to cross-examine survival data associated with the TCGA dataset. Stratification of the patients into two groups (low versus high co-expression of drug gene targets with invadopodia genes) identified a significant impact on GBM survival compared to the individual groups (Supp. Figure 1; Supp. Table 3).

**Table 1:** Invadopodia regulators are overexpressed in glioma relative to normal brain tissue

Invadopodia marker	Cancer tissue sample	Number of samples	Corresponding tissue sample	Number of samples	Total measured genes	Mean Fold Change (Log2)	*p* value	Sample type	Platform	Study
Tks4	GBM	27	Normal Brain	4	14,836	3.257	1.12E−04	mRNA	ND	Bredel Brain 2
MMP2	GBM	27	Normal Brain	4	14,836	6.426	5.41E−04	mRNA	ND	Bredel Brain 2
Nck1	GBM	27	Normal Brain	4	14,836	1.717	1.00E−02	mRNA	ND	Bredel Brain 2
Tks5	GBM	27	Normal Brain	4	14,836	1.399	0.048	mRNA	ND	Bredel Brain 2
MMP2	AO	6	Normal Brain	4	14,836	2.636	1.60E−02	mRNA	ND	Bredel Brain 2
Tks5	AO	3	Normal Brain	4	14,836	1.593	0.045	mRNA	ND	Bredel Brain 2
Nck1	AA	23	Normal Brain	6	19,574	1.705	4.76E−05	mRNA	Human Genome U133 Plus 2.0 Array	French
MMP2	GBM	30	Normal Brain	3	9957	4.537	3.00E−03	mRNA	ND	Liang
Tks4	GBM	30	Normal Brain	3	9957	1.492	1.40E−02	mRNA	ND	Liang
Nck1	GBM	30	Normal Brain	3	9957	1.626	1.90E−02	mRNA	ND	Liang
Tks4	GBM	80	Normal Brain	4	19,574	2.241	1.32E−06	mRNA	Human Genome U133 Plus 2.0 Array	Murat
MMP2	GBM	80	Normal Brain	4	19,574	2.92	2.98E−04	mRNA	Human Genome U133 Plus 2.0 Array	Murat
Nck2	GBM	80	Normal Brain	4	19,574	1.135	2.00E−03	mRNA	Human Genome U133 Plus 2.0 Array	Murat
Nck1	GBM	80	Normal Brain	4	19,574	1.885	5.00E−03	mRNA	Human Genome U133 Plus 2.0 Array	Murat
Src	GBM	80	Normal Brain	4	19,574	1.035	4.50E−02	mRNA	Human Genome U133 Plus 2.0 Array	Murat
Grb2	GBM	27	Normal Brain	7	8603	1.701	2.75E−04	mRNA	Human Genome U95A-Av2 Array	Shai
Grb2	A	5	Normal Brain	7	8603	2.169	1.49E−04	mRNA	Human Genome U95A-Av2 Array	Shai
MMP2	GBM	81	Normal Brain	23	19,574	3.548	7.99E−16	mRNA	Human Genome U133 Plus 2.0 Array	Sun
Tks4	GBM	81	Normal Brain	23	19,574	2.194	2.50E−04	mRNA	Human Genome U133 Plus 2.0 Array	Sun
Nck1	GBM	81	Normal Brain	23	19,574	1.305	5.41E−07	mRNA	Human Genome U133 Plus 2.0 Array	Sun
Src	GBM	81	Normal Brain	23	19,574	1.601	2.00E−03	mRNA	Human Genome U133 Plus 2.0 Array	Sun
N-WASP	GBM	81	Normal Brain	23	19,574	1.338	1.10E−02	mRNA	Human Genome U133 Plus 2.0 Array	Sun
MMP2	AA	19	Normal Brain	23	19,574	2.697	1.33E−05	mRNA	Human Genome U133 Plus 2.0 Array	Sun
Nck1	AA	19	Normal Brain	23	19,574	1.516	1.20E−07	mRNA	Human Genome U133 Plus 2.0 Array	Sun
N-WASP	AA	19	Normal Brain	23	19,574	1.445	2.00E−02	mRNA	Human Genome U133 Plus 2.0 Array	Sun
N-WASP	OD	50	Normal Brain	23	19,574	1.475	3.00E−03	mRNA	Human Genome U133 Plus 2.0	Sun
Src	AA	19	Normal Brain	23	19,574	1.466	4.60E−02	mRNA	Human Genome U133 Plus 2.0 Array	Sun
Tks4	AA	19	Normal Brain	23	19,574	1.704	1.30E−05	mRNA	Human Genome U133 Plus 2.0 Array	Sun
MMP2	GBM	542	Normal Brain	10	12,624	4.818	4.06E−10	mRNA	Human Genome U133A Array	TCGA
Nck1	GBM	542	Normal Brain	10	12,624	2.056	4.06E−09	mRNA	Human Genome U133A Array	TCGA
Cortactin	GBM	542	Normal Brain	10	12,624	1.353	0.003	mRNA	Human Genome Y133A Array	TCGA

mRNA expression levels of invadopodia regulators were examined in GBM and normal brain tissue within the Oncomine database. Displayed in this table are the mean fold changes versus normal brain in each study and overall *p* value in that dataset. Gene expression data are log transformed and normalized as previously described

*ND* not defined

**Table 2:** Bortezomib, Everolimus and Fludarabine gene targets are overexpressed in glioma

Gene	Cancer tissue sample	Number of samples	Corresponding tissue sample	Number of samples	Total measured genes	Mean fold change (Log2)	*p* value	Sample type	Platform	Study
Bortezomib										
NFKB1	AOA	6	Normal Brain	4	14,836	1.413	0.041	mRNA	ND	Bredel Brain 2
NFKB1	A	45	Normal Brain	6	5338	1.594	0.018	mRNA	Human Gene FL Array	Rickman Brain
NFKB1	GBM	27	Normal Brain	4	14,836	1.778	1.39E−04	mRNA	ND	Bredel Brain 2
NFKB1	GBM	30	Normal Brain	3	9957	1.301	0.043	mRNA	ND	Liang Brain
NFKB1	GBM	80	Normal Brain	4	19,574	1.362	2.55E−04	mRNA	Human Genome U133 Plus 2.0 Array	Murat Brain
NFKB1	GBM	27	Normal Brain	7	8603	1.281	0.021	mRNA	Human Genome U95-Av2 Array	Shai Brain
NFKB1	GBM	81	Normal Brain	23	19,574	1.166	0.004	mRNA	Human Genome U133 Plus 2.0 Array	Sun Brain
NFKB1	GBM	542	Normal Brain	10	12,624	2.048	4.43E−09	mRNA	Human Genome U133A Array	TCGA Brain
NOXA	AA	19	Normal Brain	23	19,574	1.648	0.006	mRNA	Human Genome U133 Plus 2.0 Array	Sun Brain
NOXA	A	44	Normal Brain	6	5338	3.695	0.031	mRNA	Human Gene FL Array	Rickman Brain
NOXA	DA	7	Normal Brain	23	19,574	3.352	0.002	mRNA	Human Genome U133 Plus 2.0 Array	Sun Brain
NOXA	GBM	80	Normal Brain	4	19,574	1.378	3.81E−05	mRNA	Human Genome U133 Plus 2.0 Array	Murat Brain
NOXA	GBM	81	Normal Brain	23	19,574	1.643	0.005	mRNA	Human Genome U133 Plus 2.0 Array	Sun Brain
NOXA	GBM	542	Normal Brain	10	12,624	1.613	1.71E−08	mRNA	Human Genome U133A Array	TCGA Brain
NOXA	OD	50	Normal Brain	23	19,574	1.59	0.005	mRNA	Human Genome U133 Plus 2.0 Array	Sun Brain
AKT	GBM	542	Normal Brain	10	12,624	1.534	3.02E−13	mRNA	Human Genome U133A Array	TCGA Brain
AKT	AOD	3	Normal Brain	4	14,836	1.856	0.001	mRNA	ND	Bredel Brain 2
AKT	OD	5	Normal Brain	4	14,836	1.450	0.005	mRNA	ND	Bredel Brain 2
AKT	GBM	27	Normal Brain	4	14,836	1.183	0.012	mRNA	ND	Bredel Brain 2
AKT	PA	8	Normal Brain	3	8603	1.579	0.017	mRNA	Human Genome U95A-Av2 Array	Gutmann Brain
AKT	A	45	Normal Brain	6	5338	1.107	0.015	mRNA	Human Gene FL Array	Rickman Brain
AKT	GBM	27	Normal Brain	7	8603	1.359	6.91E−4	mRNA	Human Genome U95A-Av2 Array	Shai Brain
AKT	GBM	81	Normal Brain	23	19,574	1.305	1.90E−7	mRNA	Human Genome U133 Plus 2.0 Array	Sun Brain
AKT	OD	50	Normal Brain	23	19,574	1.205	7.81E−6	mRNA	Human Genome U133 Plus 2.0 Array	Sun Brain
AKT	AOD	23	Normal Brain	6	19,574	1.541	1.50E−4	mRNA	Human Genome U133 Plus 2.0 Array	French Brain
AKT	GBM	80	Normal Brain	4	19,574	1.431	4.01E−4	mRNA	Human Genome U133 Plus 2.0 Array	Murat Brain
Everolimus										
MTOR	A	5	Normal Brain	7	8603	1.452	0.004	mRNA	Human Genome U95-AVr Array	Shai Brain
MTOR	DA	7	Normal brain	23	19,574	2.094	0.005	mRNA	Human Genome U133 Plus 2.0 Array	Sun Brain
MTOR	GBM	22	Normal Brain	3	19,574	2.809	9.26E−07	mRNA	Human Genome U133 Plus 2.0 Array	Lee Brain
MTOR	GBM	27	Normal Brain	7	8603	1.292	2.37E−04	mRNA	Human Genome U95-Av2 Array	Shai Brain
MTOR	GBM	81	Normal Brain	23	19,574	1.777	0.002	mRNA	Human Genome U133 Plus 2.0 Array	Sun Brain
Fludarabine										
RRM1	A	5	Normal Brain	7	8603	1.146	0.031	mRNA	Human Genome U95-Av2 Array	Shai Brain
RRM1	OD	3	Normal Brain	3	8603	1.394	0.03	mRNA	Human Genome U95-Av2 Array	Shai Brain
RRM1	GBM	27	Normal Brain	7	8603	1.494	1.64E−05	mRNA	Human Genome U95-Av2 Array	Shai Brain
RRM1	A	45	Normal Brain	6	5338	2.913	0.002	mRNA	Human Gene FL Array	Rickman Brain
RRM1	GBM	30	Normal Brain	3	9957	1.368	5.50E−04	mRNA	ND	Liang Brain
RRM1	GBM	80	Normal Brain	4	19,574	2.014	3.58E−05	mRNA	Human Genome U133 Plus 2.0 Array	Murat Brain
RRM1	OD	50	Normal Brain	23	19,574	1.442	7.37E−05	mRNA	Human Genome U133 Plus 2.0 Array	Sun Brain
RRM1	AA	19	Normal Brain	23	19,574	1.672	2.61E−05	mRNA	Human Genome U133 Plus 2.0 Array	Sun Brain
RRM1	GBM	81	Normal Brain	23	19,574	2.013	2.21E−11	mRNA	Human Genome U133 Plus 2.0 Array	Sun Brain
RRM1	GBM	542	Normal Brain	10	12,624	3.231	9.25E−10	mRNA	Human Genome U133A Array	TCGA Brain
RRM1	AOD	23	Normal Brain	6	19,574	2.302	1.18E−06	mRNA	Human Genome U133 Plus 2.0 Array	French Brain
RRM1	AOA	4	Normal Brain	6	19,574	2.011	6.17E−05	mRNA	Human Genome U133 Plus 2.0 Array	French Brain
POLA1	AA	19	Normal Brain	23	19,574	1.665	7.69E−10	mRNA	Human Genome U133 Plus 2.0 Array	Sun Brain
POLA1	GBM	81	Normal Brain	23	19,574	1.492	5.70E−09	mRNA	Human Genome U133 Plus 2.0 Array	Sun Brain
POLA1	QD	50	Normal Brain	23	19,574	1.338	1.52E−05	mRNA	Human Genome U133 Plus 2.0 Array	Sun Brain
POLA1	DA	7	Normal Brain	23	19,574	1.264	7.40E−02	mRNA	Human Genome U133 Plus 2.0 Array	Sun Brain
POLA1	GBM	80	Normal Brain	4	19,574	1.9982	8.69E−08	mRNA	Human Genome U133 Plus 2.0 Array	Murat Brain
POLA1	GBM	27	Normal Brain	7	8603	1.394	8.35E−04	mRNA	Human Genome U95-Av2 Array	Shai Brain
POLA1	OD	3	Normal Brain	7	8603	1.585	1.00E−02	mRNA	Human Genome U95-Av2 Array	Shai Brain
POLA1	AA	5	Normal Brain	7	8603	1.233	2.30E−02	mRNA	Human Genome U95-Av2 Array	Shai Brain
POLA1	GBM	542	Normal Brain	10	12,624	1.756	1.79E−07	mRNA	Human Genome U133A Array	TCGA
POLA1	AOA	4	Normal Brain	6	19,574	1.389	1.40E−02	mRNA	Human Genome U133 Plus 2.0 Array	French Brain
POLA1	AOD	23	Normal Brain	6	19,574	1.303	0.013	mRNA	Human Genome U133 Plus 2.0 Array	French Brain
DCK	OD	3	Normal Brain	7	8603	1.426	9.48E−04	mRNA	Human Genome U95A-Av2 Array	Shai Brain
DCK	A	5	Normal Brain	7	8603	1.274	1.00E−02	mRNA	Human Genome U95A-Av2 Array	Shai Brain
DCK	GBM	542	Normal Brain	10	12,624	1.162	2.80E−02	mRNA	Human Genome U133A Array	TCGA Brain

mRNA expression levels of bortezomib, everolimus and fludarabine gene targets regulators was examined in glioma and normal brain tissue within the Oncomine database. Displayed in this table are the mean fold changes versus normal brain in each study and overall *p* value in that dataset. Gene expression data are log transformed and normalized as previously described

*ND* not defined

### STRING Protein–protein analysis indicates potential interactions between invadopodia regulators and drug targets

A protein–protein interaction (PPI) network was constructed for the FDA-approved drug target genes and the invadopodia regulator genes. As can be seen in Supp. Figure 2a, experimental and curated database evidence suggest that there are protein–protein interactions between a number of the invadopodia regulators and the drug targets. The known interactions with drug targets include NFKB1 and AKT1 (bortezomib) and MTOR (Everolimus). The corresponding confidence PPI network map (Supp. Figure 2b) shows prominent interactions between the drug target genes and the invadopodia regulators Src and Grb2, which is supported by the ‘combined score’ (Supp. Figure 2c) as determined by the STRING network pipeline.

## Discussion

Despite advances in medical technology and decades of research and numerous clinical trials [30], the prognosis for GBM patients still remains poor [4, 30], with the highly infiltrative phenotype of GBM cells contributing to tumour recurrence [33]. We have previously demonstrated the presence of invadopodia in primary tumour cells isolated from ex-vivo cultured GBM specimens [7] and that the formation and activity of invadopodia coupled with enhanced MMP-2 secretion is observed in GBM cells surviving RT and TMZ treatment [34]. The current standard of care for GBM patients includes fractionated radiotherapy at 2 Gy per day [30] and it has been previously determined that the concentration of TMZ in the CSF of GBM patients can range between 5 and 50 μM [35]. Therefore, in our current study, we used a radiotherapy dose of 2 Gy and TMZ concentration of 50 μM and observed enhanced invadopodia-mediated FITC-gelatin degrading activity in the LN229 GBM cells, which supported our previous findings at higher doses of RT and TMZ [34]. This highlights the urgent need for exploring therapeutic strategies to combat invadopodia formation in GBM cells that survive RT/TMZ treatment, which formed the basis for evaluating the FDA-approved agents in our study.

Importantly, previous studies have demonstrated that these three drugs can reduce the invasive ability of other cancer cell types, but these observations were not linked to a decrease in the matrix degrading ability of invadopodia. Bortezomib treatment reduces the migration and invasive capacity of a cervical carcinoma HeLa cell line, which was associated with a decrease in the intracellular expression of MMP2 and MMP9 [36], and this is also observed in bortezomib treated chondrosarcoma cells [37]. Significant inhibition of the invasive capacity of human breast cancer cells [38] and ovarian cancer cells [39] in vitro, coupled with a decrease in MMP9 expression has also been observed with everolimus treatment. Notably, the in vitro based matrix invasive capacity of colorectal cancer cells is also reduced with fludarabine treatment [40]. These observations support the findings from this current study demonstrating that these FDA-approved agents can reduce the invasive capacity of GBM cells by impacting invadopodia activity.

Fludarabine is a chemotherapeutic agent used for treating haematological malignancies due to its ability in disrupting DNA synthesis [41]. Early studies in the late 1980’s, evaluating the efficacy of fludarabine in glioma patients concluded that it was not an effective therapeutic agent at the utilized dosages (18 or 25 mg/m^2^/day) [42, 43] and recommended that further study of the drug was not warranted. However, these were small trials (23 patients [42] and 15 patients [43], respectively) involving recurrent anaplastic astrocytoma or glioblastoma patients. However, one trial did report positive responses in some patients (partial responder—1; improved responder—2; stable disease—2) [42]. Also, a recent study has since demonstrated that fludarabine phosphate can inhibit inositol-requiring enzyme 1 (IRE1) activity, which in turn sensitizes GBM cells to TMZ treatment [44] and therefore could potentially be integrated with the current Stupp based protocol prior to TMZ administration, to improve patient outcome.

Mammalian target of rapamycin (mTOR) signalling plays a critical role in cellular functions for normal and cancer cells. The mTOR multiprotein complex, mTOR1 is significantly deregulated in cancer including GBM [45], which has driven an increasing interest in rapamycin-based therapies including everolimus (RAD001). There have been varied outcomes from everolimus trials for glioma patients, but a phase II trial of recurrent low grade glioma patients resulted in a high degree of disease stability [46]. The median survival of newly diagnosed GBM patients treated with a combination of everolimus and conventional chemoradiation is comparable to contemporary studies, but inferior to controls in a randomized study [47]. However, Babak and Mason [48] identified that 77.1% of patients in the control arm of this study received adjuvant TMZ, whereas only 60.2% in the everolimus arm received TMZ. This may have had an unfavourable impact on survival, especially since the hypermethylation of O6-methylguanine-DNA methyltransferase would have conferred a survival advantage in the control arm. These contributing factors must be considered when further examining mTOR inhibition-based therapy in future trials, especially as mTORC1, as a potential therapeutic target is overexpressed in GBM.

Bortezomib is a dipeptide boronic acid derivative and a reversible inhibitor of the 26S proteasome. It has been particularly successful in the treatment of myeloma, as it induces death in multiple myeloma cells at doses that are non-toxic to normal blood peripheral cells with therapeutic efficacy [49]. Promising clinical results have been observed in the treatment of solid cancers [50–56]. Phase I and II clinical trials evaluating bortezomib treatment of GBM patients have shown that the combination with radiotherapy and TMZ is well tolerated [57–59], and that stable clinical symptoms and radiological response and improvements in survival compared to historical controls appear promising [57, 59].

In a recent, phase II study [57], the addition of bortezomib to radiotherapy and TMZ resulted in a median overall survival of 19.1 months and a pronounced median overall survival of 61 months in MGMT-methylated GBM patients versus 16.4 in unmethylated patients. Whilst this is only a small study, the encouraging positive results warrant further trials investigating the combination of radiotherapy, TMZ and bortezomib. Importantly, it has been observed to cross the blood brain barrier (BBB) in humans and mice [59, 60]. However, some phase II clinical trials investigating the efficacy of systemically administered bortezomib administered with tamoxifen [61] or vorinostat [62] demonstrated no therapeutic benefit, indicating potentially a low CNS penetrance due to the BBB. But, it has been proposed that efflux transporters such as P-glycoprotein (P-gp, ABCB1), function to intercept drugs entering the CNS capillary cells and transport them back into the blood [63]. By inhibiting drug efflux with the use of ABC transporter inhibitors, the CNS penetrance of drugs such as bortezomib can potentially be improved [64]. The intratumoural administration of bortezomib into the cranial cavity has also been proposed as an effective therapy for GBM, based on osmotic pump administration of the drug in a mouse glioma model [65].

The predicted BBB penetrance of the candidate drugs based on physiochemical properties as determined by ADMET (absorption, distribution, metabolism, excretion, and toxicity) [66] is displayed in Supp. Table 4, indicating that everolimus and fludarabine, are predicted to achieve higher BBB penetrance than bortezomib. However, due to the multiple physicochemical and biological factors that can contribute to the permeability of the BBB, it is difficult to accurately predict BBB penetrance based on these factors alone [67]. Exosomes which are secreted by cancer and normal cells, facilitate intercellular communication through the transfer of functional cargo (mRNA, miRNA, proteins). They have been demonstrated to cross the BBB [68] and are being investigated as therapeutic vehicles for pharmaceuticals or small interfering RNA (siRNA) in cancer treatment and incorporation into the clinical setting [69].

As a drug delivery system, exosomes display a high degree of biocompatibility and reduced clearance rate compared to direct systemic administration of chemotherapeutic agents, prompting investigation of the exosomal encapsulation of drugs such as doxorubicin and paclitaxel [68, 70]. This approach has been proposed for the delivery of TMZ to GBM cells compared to a systemic approach to reduce systemic metabolism as a ‘non-encapsulated drug’ and decreasing the observed off-target effects associated with systemically administered chemotherapeutics [71].If the candidate drugs, bortezomib, everolimus and fludarabine could be encapsulated within exosomes, BBB penetration and local intratumoural concentration maybe increased.

While inhibition of the 26S proteasome is the main mechanism of action for bortezomib, multiple mechanisms may contribute to the therapeutic action of bortezomib. This may include the upregulation of a proapoptotic protein, phorbol-12-myristate-13-acetate-induced protein 1 (NOXA), which may in turn interact with the anti-apoptotic proteins of the Bcl-2 subfamily, Bcl-XL and Bcl-2, resulting in the apoptotic death of the tumour cells. Also, amplification of the epidermal growth factor (EGFR) and loss of phosphatase and tensin homolog (PTEN) can contribute to the malignant phenotype of glioma [72] and the downstream targets, Akt and NF-kB, impact oncogenesis, cell proliferation and apoptosis [73]. A study by Bredel et al., identified that NFκBIA deletion in GBM is a negative prognostic marker and that inhibition of NFκB by bortezomib may be beneficial [74].

The data presented from our current study demonstrates that FDA-approved drugs not initially designed for use in the treatment of GBM patients have the potential to be repurposed, as dual ‘chemotherapeutic’ and ‘anti-invasive’ (anti-invadopodia) agents targeting GBM cells that survive RT and TMZ. The three candidate FDA-approved drugs, bortezomib, everolimus and fludarabine all displayed a dual cytotoxic and anti-invadopodia effect, especially reducing RT/TMZ treatment-induced invadopodia activity. Further research investigating the use of these three agents, as an adjuvant treatment in targeting the invasive capacity of GBM cells that survive RT/TMZ treatment of GBM patients is warranted.

## Supplementary Information

Below is the link to the electronic supplementary material.

Supplementary file1 (TIF 172535 KB)

Supplementary file2 (TIF 43194 KB)

Supplementary file3 (DOCX 14 KB)

Supplementary file4 (DOCX 17 KB)

Supplementary file5 (DOCX 15 KB)

Supplementary file6 (DOCX 15 KB)

## Data Availability

There is no associated data to be made available.

## Abbreviations

A: Astrocytoma
AA: Anaplastic Astrocytoma
AO: Anaplastic Oligodendroglioma
AOA: Anaplastic Oligoastrocytoma
AOD: Anaplastic Oligodendroglioma
AKT1: AKT Serine/Threonine Kinase 1
AO: Anaplastic Oligoastrocytoma
BBB: Blood Brain Barrier
CNS: Central nervous system
CO_2_: Carbon dioxide
CSF: Cerebrospinal fluid
DA: Diffuse Astrocytoma
DAPI: 4′,6-Diamidino-2-phenylindole
DCK: Deoxycytidine Kinase
DMSO: Dimethyl sulphoxide
ECM: Extracellular matrix
EGFR: Epidermal growth factor receptor
FDA: U.S. Food and Drug Administration
FITC: Fluorescein isothiocyanate
GAPDH: Glyceraldehyde 3-phosphate dehydrogenase
GBM: Glioblastoma
Gy: Gray
Grb2: Growth factor receptor-bound protein 2
kDa: Kilodalton
MGMT: O-6-methylguanine-DNA methyltransferase
ml: Millilitres
MMP: Matrix-metalloproteinase
MMP-2: Matrix-metalloproteinase-2
MMP-9: Matrix-metalloproteinase-9
mRNA: Messenger RNA
MT1-MMP: Membrane type 1-matrix metalloproteinase
μg: Microgram
μM: Micromolar
MTOR: Mechanistic Target Of Rapamycin
Nck1: NCK Adaptor Protein 1
NFKB1: Nuclear Factor Kappa B Subunit 1
nm: Nanometre
NOXA1: NADPH Oxidase Activator 1
N-WASP: Neural Wiskott-Aldrich syndrome protein
OD: Oligodendroglioma
PBS: Phosphate buffered saline
POLA1: DNA Polymerase Alpha 1, Catalytic Subunit
RRM1: Ribonucleotide Reductase Catalytic Subunit M1
RT: Radiotherapy
TBST: Tris Buffered Saline with Tween 20
TCGA: The Cancer Genome Atlas
Tks4: Tyrosine kinase substrate with 4 SH3 domains
Tks5: Tyrosine kinase substrate with 5 SH3 domains
TMZ: Temozolomide
VEGFR: Vascular Endothelial Growth Factor
Vol: Volume
