# Supplementary figures and images for: Repurposing FDA-approved drugs as inhibitors of therapy-induced invadopodia activity in glioblastoma cells
Source: Mol Cell Biochem. 2022 Oct 27;478(6):1251–67. doi: 10.1007/s11010-022-04584-0 (PMC10164021; doi:10.1007/s11010-022-04584-0)

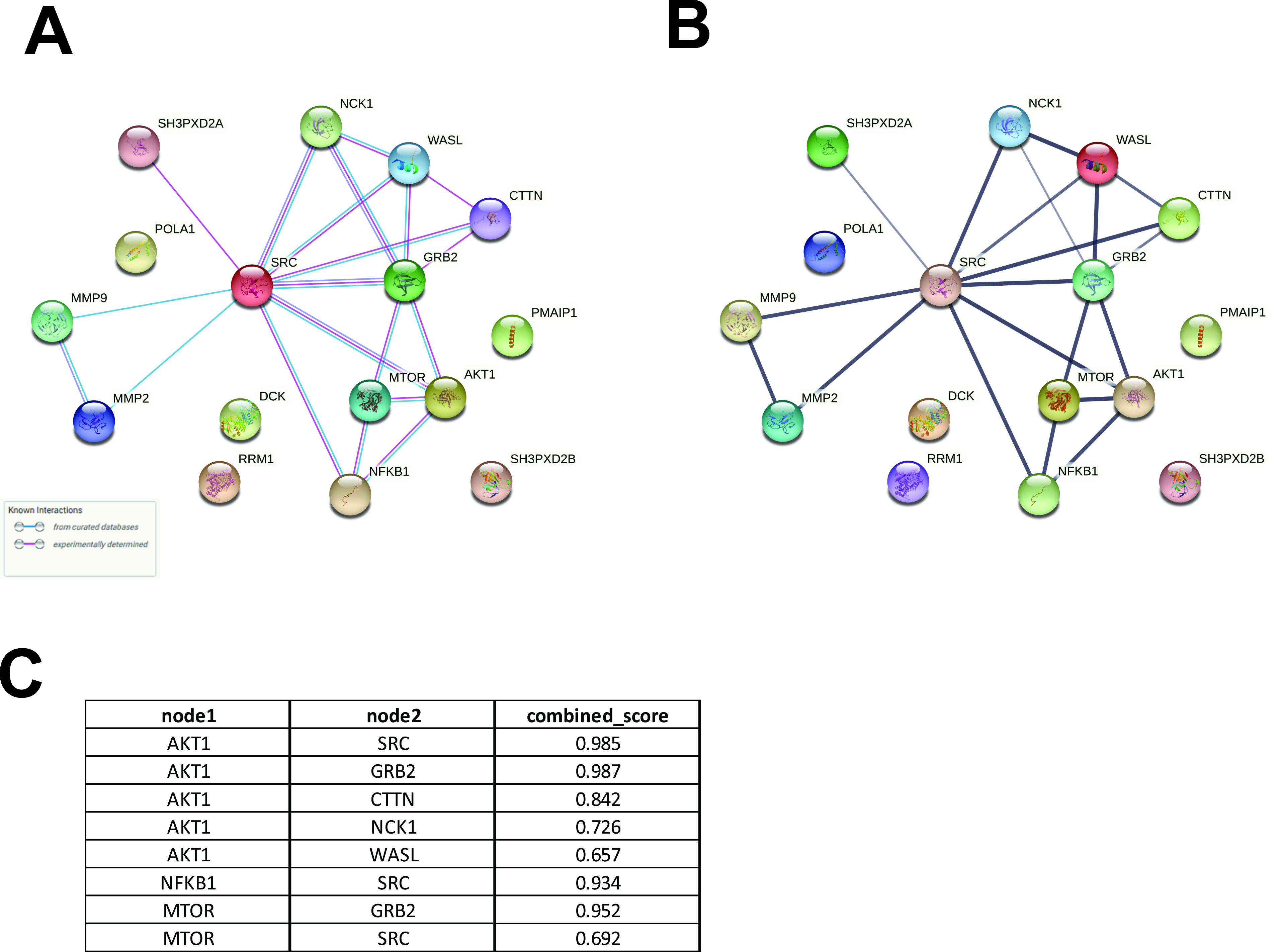

Supplement: Supplementary file 2 — Supplementary file2 (TIF 43194 KB) [file 11010_2022_4584_MOESM2_ESM.tif]
