# Supplementary material for: Repurposing FDA-approved drugs as inhibitors of therapy-induced invadopodia activity in glioblastoma cells
Source: Mol Cell Biochem. 2022 Oct 27;478(6):1251–67. doi: 10.1007/s11010-022-04584-0 (PMC10164021; doi:10.1007/s11010-022-04584-0)
Supplement: Supplementary file 3 — Supplementary file3 (DOCX 14 KB) [file 11010_2022_4584_MOESM3_ESM.docx]

**Supplementary Table 1** FDA-Approved Drugs utilized in this study

| Drug | Known Target |
| --- | --- |
| Axitinib | VEGFR and PDGFR |
| Bortezomib (Velcade) | Proteasome |
| Crizotinib (PF-02341066) | c-Met |
| Dequalinium Chloride | PKC |
| Everolimus (RAD001) | mTOR |
| Fludarabine (Fudara) | STAT, DNA/RNA synthesis |
| Imatinib Mesylate | PDGFR, c-Kit, Bcr-Abl |
| Irinotecan | Topoisomerase |
| Lapatinib (GW572016 | EGFR, HER2 |
| 2-Methoxyestradiol (2ME2) | HIF |
| Nilotinib (AMN-107) | BCR-Abl |
| Pazopanib Hcl | VEGFR and PDGFR |
| Pimecrolimus | Inflammatory cytokines |
| Rosiglitazone (Avandia) | PPAR |
| Rosiglitazone HCl | PPAR |
| Rosiglitazone maleate | PPAR |
| Temsirolimus (Torisel) | mTOR |
| Tofacitinib citrate (CP-690550 citrate) | JAK |
| Vismodegib (GDC-0449) | Hedgehog |
| Vorinostat (SAHA) | HDAC |
