# Supplementary material for: Repurposing FDA-approved drugs as inhibitors of therapy-induced invadopodia activity in glioblastoma cells
Source: Mol Cell Biochem. 2022 Oct 27;478(6):1251–67. doi: 10.1007/s11010-022-04584-0 (PMC10164021; doi:10.1007/s11010-022-04584-0)
Supplement: Supplementary file 4 — Supplementary file4 (DOCX 17 KB) [file 11010_2022_4584_MOESM4_ESM.docx]

**Supplementary Table 2** Short-listed FDA-Approved Drugs displaying greatest reduction in GBM cell viability

| Drug | Target | Greatest Cell Viability Reduction Efficiency |
| --- | --- | --- |
| 10 µM FDA-approved drug concentration | | |
| Fludarabine | STAT, DNA/RNA synthesis | 3/3 cell lines |
| Bortezomib | Proteasome | 3/3 cell lines |
| Dequalinium Chloride | PKC | 2/3 cell lines |
| Nilitonib | BCR-Abl | 2/3 cell lines |
|  |  |  |
| 1 µM FDA-approved drug concentration | | |
| Fludarabine | STAT, DNA/RNA synthesis | 3/3 cell lines |
| Bortezomib | Proteasome | 3/3 cell lines |
| 2-methoxyestradiol | HIF | 1/3 cell lines |
| Everolimus | mTOR | 1/3 cell lines |
|  |  |  |
| 0.1 µM FDA-approved drug concentration | | |
| Fludarabine | STAT, DNA/RNA synthesis | 3/3 cell lines |
| Bortezomib | Proteasome | 3/3 cell lines |
| Everolimus | mTOR | 3/3 cell lines |
| Temsirolimus | mTOR | 1/3 cell lines |
|  |  |  |
| 0.01 µM FDA-approved drug concentration | | |
| Bortezomib | Proteasome | 3/3 cell lines |
| Everolimus | mTOR | 3/3 cell lines |
| Pazopanib HCl | VEGFR and PDGFR | 2/3 cell lines |
| Fludarabine | STAT, DNA/RNA synthesis | 2/3 cell lines |
