# Supplementary material for: Repurposing FDA-approved drugs as inhibitors of therapy-induced invadopodia activity in glioblastoma cells
Source: Mol Cell Biochem. 2022 Oct 27;478(6):1251–67. doi: 10.1007/s11010-022-04584-0 (PMC10164021; doi:10.1007/s11010-022-04584-0)
Supplement: Supplementary file 5 — Supplementary file5 (DOCX 15 KB) [file 11010_2022_4584_MOESM5_ESM.docx]

**Supplementary Table 3** Co-expression of invadopodia regulator genes and bortezomib, everolimus and fludarabine gene targets results in poorer GBM patient survival

| **Drug** | **Expression Platform** | **Microarray Platform** | **Drug Target Genes p-value** | **Invadopodia Genes p-value**  ****** | **Drug Target Genes / Invadopodia Genes p-value**  ******* |
| --- | --- | --- | --- | --- | --- |
| Bortezomib* | All^#^ | 3-platform Aggregates | 0.932 | 0.061 | 0.006 |
| Fludarabine* | All^#^ | 3-platform Aggregates | 0.145 | 0.061 | 0.002 |
| Everolimus * | All^#^ | 3-platform Aggregates | 0.256 | 0.061 | 0.016 |

*Drug target genes : Bortezomib – NFKB1, NOXA, AKT1; Fludarabine – RRM1, POLA1, DCK; Everolimus – mTOR. **Invadopodia genes – CTTN, MMP2, MMP9, NCK1, SH3PXD2A, SH3PXD2B, Src, WASL, Grb2. The analysis was performed using the Glioblastoma Bio Discovery Portal (GBM-BioDP). ***Representative Kaplan Meier curves for the Full Cohort of the combined gene groups are displayed in supplementary figure 1. ^#^Gene expression data derived from three microarray platforms (Affymetrix, Agilent, and Exon).
