# Supplementary material for: Repurposing FDA-approved drugs as inhibitors of therapy-induced invadopodia activity in glioblastoma cells
Source: Mol Cell Biochem. 2022 Oct 27;478(6):1251–67. doi: 10.1007/s11010-022-04584-0 (PMC10164021; doi:10.1007/s11010-022-04584-0)
Supplement: Supplementary file 6 — Supplementary file6 (DOCX 15 KB) [file 11010_2022_4584_MOESM6_ESM.docx]

**Supplementary Table 4** Candidate drug predicted blood-brain barrier penetrance properties

| **Drug Name** | **Water Solubility**  **ALOGPS**  **(mg/mL)** | **logP**  **ALOGPS** | **logS**  **ALOGPS** | **ADMET**  **Blood Brain Barrier (BBB)**  **Penetration Level** |
| --- | --- | --- | --- | --- |
| Bortezomib | 0.0532 | 0.89 | -3.9 | - / 0.6533 |
| Everolimus | 0.00163 | 5.01 | -5.8 | - / 0.9541 |
| Fludarabine | 12.1 | -0.62 | -1.4 | + / 0.9233 |

Candidate drug predicted blood–brain barrier penetrance properties. We examined information on the three candidate drugs, bortezomib, everolimus and fludarabine present in the online database, ‘DrugBank’(go.drugbank.com). DrugBank is a knowledge base consisting of clinical information such as side effects, drug interactions, and molecular-level data including chemical structures, predicted properties, and protein interactions. Based on their chemical structures, the table lists the predicted water solubility (as determined by ALOGPS) and the predicted blood–brain barrier penetrance (as determined by ADMET - Absorption, Distribution, Metabolism, Elimination, Toxicity), as extracted from the DrugBank database. A ‘+’ ADMET value indicates a ‘yes’ for the predicted property of blood–brain barrier penetrance. As defined by the predicted ADMET features in DrugBank, the blood–brain barrier penetrance values of 0.6533, 0.9541 and 0.9233 indicate that there is a 65.33%, 95.41% and 92.33% probability that bortezomib, everolimus and fludarabine will cross the blood–brain barrier. logP—drug lipophilicity; logS—drug water solubility.
